# Loss of *Drosophila* E3 Ubiquitin Ligase Hyd Promotes Extra Mitosis in Germline Cysts and Massive Cell Death During Oogenesis

**DOI:** 10.3389/fcell.2020.600868

**Published:** 2020-11-09

**Authors:** Natalia V. Dorogova, Yuliya A. Galimova, Elena Us. Bolobolova, Elina M. Baricheva, Svetlana A. Fedorova

**Affiliations:** ^1^Department of Cell Biology, Institute of Cytology and Genetics, SB RAS, Novosibirsk, Russia; ^2^Department of the Regulation of Genetic Processes, Institute of Molecular and Cellular Biology, SB RAS, Novosibirsk, Russia

**Keywords:** oogenesis, cell death, E3 ubiquitin ligase, germ cells, *Drosophila*, *hyperplastic disc* gene

## Abstract

The *Drosophila hyperplastic disc* (*hyd)* gene is the ortholog of mammalian tumor suppressor *EDD*, which is implicated in a wide variety of cellular processes, and its regulation is impaired in various tumors. It is a member of the highly conserved HECT family of E3 ubiquitin ligases, which directly attach ubiquitin to targeted substrates. In early works, it was shown that *Drosophila* Hyd may be a tumor suppressor because it is involved in the control of imaginal-disc cell proliferation and growth. In this study, we demonstrated that Hyd is also important for the regulation of female germ cell proliferation and that its depletion leads to additional germline cell mitoses. Furthermore, we revealed a previously unknown Hyd function associated with the maintenance of germ cells’ viability. A reduction in *hyd* expression by either mutations or RNA interference resulted in large-scale germ cell death at different stages of oogenesis. Thus, the analysis of phenotypes arising from the *hyd* deficiency points to Hyd’s role in the regulation of germline metabolic processes during oogenesis.

## Introduction

The *Drosophila melanogaster hyperplastic disc* gene (*hyd*) has been classified as a tumor suppressor gene owing to the overgrowth phenotype of imaginal disc cells in mutant backgrounds (Mansfield et al., 1994; Shearer et al., 2015). It is an ortholog of mammalian *EDD*, which was originally identified as a progestin-regulated gene in human T47D breast cancer cells (*EDD* stands for ‘E3 identified by differential display’) (Callaghan et al., 1998; Clancy et al., 2003). Impaired regulation of *EDD* contributes to tumorigenesis, and various alterations of the *EDD* locus (mutations, deletions and especially amplifications) have been found in a number of common human cancers. The *hyd* gene, just as *EDD*, encodes a large protein (approximately 300 kDa) that is a member of the highly conserved family of E3 ubiquitin ligases containing the HECT domain, which mediates specific recognition of substrate proteins and their targeting by ubiquitin (Scheffner et al., 1995; Metzger et al., 2012). Ubiquitination affects the fate and properties of proteins: from proteasomal degradation to changes of a functional activity in such processes as DNA repair, transcription, immunity, autophagy and protein sorting and trafficking (Bhoj and Chen, 2009; Dikic et al., 2009). Through ubiquitination of a wide variety of substrate proteins, E3 ubiquitin ligases participate in the regulation of diverse cellular processes. Dysregulation or dysfunction of E3 ubiquitin ligases gives rise to abnormalities in the ubiquitination system and causes serious pathologies, including neurodegeneration and cancer (Shearer et al., 2015).

E3 ubiquitin ligase Hyd is involved in numerous biological processes and is necessary at all stages of *Drosophila* development. Null-alleles of *hyd* are lethal at the larval stage, and temperature-sensitive alleles induce imaginal-disc hyperplasia and adult gonadal defects leading to sterility (Martin et al., 1977; Mansfield et al., 1994). A clone analysis has revealed that Hyd negatively regulates the expression of *Hedgehog (Hh)* and *Decapentaplegic (Dpp)* in the eye and wing discs. Loss of *hyd* function induces ectopic expression of both genes, resulting in disc cell overgrowth (Lee et al., 2002; Wang et al., 2014). These data have allowed researchers to conclude that Hyd is involved in the regulation of cell proliferation and cell cycle control through Hh signaling. In spermatogenesis, however, Hyd does not manifest the tumor suppressor properties; *hyd* mutants show substantial structural abnormalities in mitosis and meiosis, without excessive cell proliferation. On the contrary, testes of the mutant males are small and contain fewer germ cells as compared to the wild type (Pertceva et al., 2010). In the present work, we continued the study on the biological functions of *hyd* in *Drosophila* oogenesis. According to previous works, *hyd*-mutant females are sterile and have defects in ovary formation and germ tissue morphology. It remains unclear what cellular event or process in oogenesis is primarily affected by the Hyd deficiency.

The *Drosophila* ovary consists of 15–20 ovarioles, which represent a chain of progressively developing egg chambers (EChs). The anterior region of each ovariole is the germarium, which includes stem cells and early germline cells (GCs) going through a series of mitotic divisions. In the posterior of the germarium, 16 daughter GCs are surrounded by a monolayer of somatic follicle cells creating an ECh. After leaving the germarium, the ECh moves along the ovariole, gradually developing into a mature oocyte. According to the size and morphology of the ECh, oogenesis can be roughly divided into 14 stages (King, 1957; Spradling, 1993). Developmentally programmed cell death in the female germline occurs at three specific stages: in newly formed cysts (region 2 of the germarium), during mid-oogenesis (stages 7–8), and during late oogenesis (stages 12–13). Under normal nutritional conditions, cell death in the germarium and at stages 7–9 (called ‘checkpoints’ of cell death in oogenesis) takes place in response to developmental abnormalities and increases dramatically under the influence of various stressors. The death of nurse cells in late oogenesis occurs as part of normal development of each egg (Jenkins et al., 2013; Peterson et al., 2015; Bolobolova et al., 2020).

Here, we present a study of the Hyd function in *Drosophila* oogenesis. We showed that a reduction in *hyd* expression by mutations or RNA interference yields two main phenotypes of the germline. The first one is manifested as extra mitotic divisions that form EChs with 32 or 64 germ cells. Nonetheless, such an effect was observed only in *hyd*-mutant flies and at a low frequency. The second phenotype was large-scale germ cell death and ECh degradation. This phenotype was strong and highly penetrant and was observed both in the mutants and after a *hyd* knockdown via RNA interference (*hyd*-RNAi). We believe that the function of Hyd in oogenesis is not related to the regulation of cell death directly. Being an E3 ubiquitin ligase, it is involved in ubiquitination of the protein substrates responsible for the metabolism and biosynthetic activity of GCs. A dysfunction of this mechanism probably causes cell death due to a deficit of resources.

## Materials and Methods

### Fly Stocks

All *Drosophila* stocks were raised at 25°C on standard cornmeal medium. We used Bloomington stocks: *kni^*ri–*1^ hyd^15^ e^1^/TM3, Sb^1^* (3718) and *y^1^ w^1118^; PBac{3HPy* + *}hyd^C017^/TM3, Sb^1^ Ser^1^* (16256) as a source of mutant alleles, *w1118; P{w* + *mC* = *PTT-GA}Pabp2* GFP protein trap strain was used as a germline nuclei marker: (48-1) kindly provided by A. Debec (Université Pierre et Marie Curie, France).

We used the following strains for RNA interference: *hyd*-RNAi -y ^1^sc^∗^v^1^;P{TRiP.HMS00343}attP2 (32352); *osk*-*GAL4* - w^1118^; P{osk-GAL4:VP16}F/TM3, Sb^1^ (44242); *nos-GAL4* - w^1118^; P{w^+mC^ = GAL4:VP16-nos.UTR}CG6325^MVD1^ (4937); *7023-GAL4* – y^1^ w^∗^; P{GawB}109-30/CyO GAL4 (7023); *7024-GAL4* – y^1^ w^∗^; P{w + mW. hs = GawB}109-39/TM3,Sb^1^ (7024); *36287-GAL4* 36287 w^∗^; P{w^+mW.hs^ = GawB}GR1.

### RNA Isolation and Quantitative Real-Time PCR

Levels of *hyd* mRNA were assigned using method of qPCR. Total RNA preparation, reverse transcription (RT) and qPCR analyses were performed using Trizol (Invitrogen), Superscript III (Invitrogen), and SYBR green kits (Syntol) following the manufacturer’s recommendations. The following primers were used: *hyd*-specific RT-HYD-Fw-5′-ACGCCAGGATTTGGTTTA CTT and RT-HYD-Rev-5′-TTCGCAGTCGGTAGATGGGA, *RpL32*-specific RPL32-F-5′-TAAGCTGTCGCACAAATGG and RPL32-R-5′-AGGAACTTCTTGAATCCGGTG as a reference gene (Yang et al., 2013). The mean values ± standard deviation of the transcript abundance of three separate determinations.

### Electron and Fluorescence Microscopy

Experimental procedures for electron and fluorescence microscopy were performed as described earlier (Pertceva et al., 2010). The primary antibodies were: monoclonal rabbit anti-VASA antibodies from Santa Cruz Biotechnology (1:300), rabbit anti-Dcp-1 (Asp216) from Cell Signaling Technology. The secondary antibodies were goat anti-rabbit conjugated to AlexaFluor-488 (1:500; Invitrogen #A-11001). TRITC-labeled phalloidin (Sigma-Aldrich #P1951) was used at 1:100 dilution to visualize F-actin as described previously (Guild et al., 1997). The LysoTracker assay was performed as described in Dorogova et al. (2014) (LysoTracker red DND-99 (Invitrogen, Molecular Probes, Basel, Switzerland). Ovaries were embedded with ProLong Gold anti-fade reagent with DAPI. Images were obtained using an AxioImager Z1 microscope with ApoTome attachment (Zeiss), AxioCam MR and AxioVision software (Zeiss, Germany).

## Results

### A Decrease in *hyd* Expression Leads to Both Ovary Atrophy and Extra Mitoses in Germline Cysts

To investigate the effect of the Hyd protein on oogenesis, we selected mutant alleles *hydC017* and *hyd15*, which have been described previously (Mansfield et al., 1994; Pertceva et al., 2010). The *hyd15* mutation, when homozygous, is lethal at the third instar larval stage, whereas *hydC017* homozygotes are viable but females exhibit a significant decline of fertility and of egg production. For cytological analysis of the effect of *hyd* mutation, viable females carrying hetero-allelic combination *hydC017/hyd15* were used. Wild-type strain—*Oregon R* and heterozygous siblings—*hyd^15^ e^1^/TM3, Sb^1^ and hyd^*C*017^/TM3, Sb^1^ Ser^1^*were taken as control.

**FIGURE 1:**
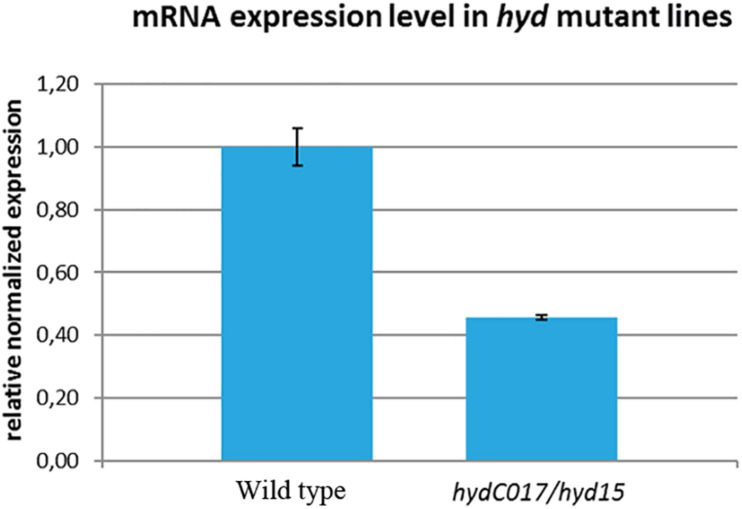
Comparison of *hyd* mRNA expression levels between wild-type and *hydC017/hyd15* ovaries as estimated by real-time PCR. The *hyd* expression is ∼2-fold lower in the mutants than in the wild-type ovaries. The mean values ± standard deviation of the transcript abundance of three separate determinations.

To determine how this hetero-allelic combination affects the level of *hyd* expression, we performed a real-time PCR analysis of adult *hydC017/hyd15* ovaries. Our data indicated that *hyd* mRNA expression was statistically significantly ∼2-fold lower in the mutants than in the control (wild type; Figure 1). Consequently, the oogenesis aberrations identified in *hydC017/hyd15* flies (as described below) could be associated with a deficiency in the *hyd* gene product.

**FIGURE 2:**
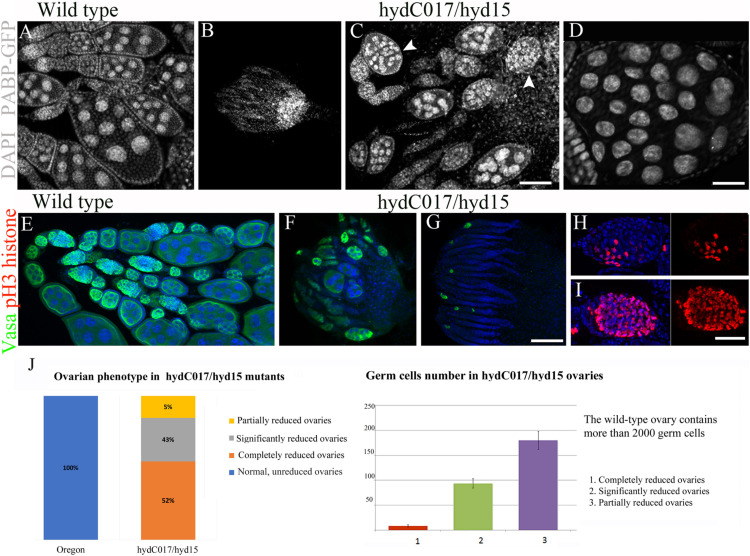
Abnormalities of oogenesis and ovary formation in *hydC017/hyd15* females. **(A)** Normal ovarian morphology in wild-type females. **(B)** A reduced ovary of a *hydC017/hyd15* female without ovarioles and EChs. **(C)** A *hydC017/hyd15* female ovary with few ovarioles containing multi-nuclear EChs (arrowheads). **(D)** A multi-nuclear ECh in a *hydC017/hyd15* ovary. **(E–G)** Germ cell visualization by anti-Vasa staining in germaria and EChs. **(E)** Wild-type ovary. **(F,G)** Germline loss in a *hydC017/hyd15* female ovary. **(H,I)** Visualization of mitotic chromosomes in germline cysts using an anti-p-H3 histone antibody. Anaphase chromosomes in the wild type **(H)** and an excessive number of anaphase chromosomes in a *hydC017/hyd15* germline cyst **(I)**. (**J**) Phenotypic variability of reduced ovaries in *hydC017/hyd15* mutants and quantification of the germ cell numbers in the *hydC017/hyd15* ovaries. The number of germ cells per ovary was determined by counting VASA-positive cells from optical serial sections, 30 ovaries for each phenotype were examined. Data represent mean ± s.d., Student’s *t*-test. For phenotypic analysis, the number of ovaries viewed—100. DNA: DAPI (blue), anti-Vasa staining: red, p-H3 histone staining: green. Scale bars: 10 μm **(A–C)**, 3 μm **(D)**, 10 μm **(E–G)**, 2 μm **(H,I)**

The *hydC017/hyd15* females were found to be sterile and laid only a single egg, which did not develop. The gonads in such females mainly contained reduced ovarioles with a small number of EChs or without them at all (Figures 2A–C). Depending on the degree of the germ-line atrophy, we have identified 3 types of ovaries: completely reduced, significantly reduced and partially reduced. Completely reduced ones had no or a few GCs. Significantly reduced ovaries contained single GCs in germaria and single EChs in ovarioles. Partially reduced ovaries contained not only atrophied, but also normal ovarioles with EChs at different stages of oogenesis. In the wild type, ovaries with any of these phenotypes were not found (Figure 2J). Partially reduced ovaries also contained EChs with an excessive GC number: 2–3-fold higher than normal (Figures 2C,D and Supplementary Table 1).

Immunostaining of a GC marker, the Vasa protein, showed that most of the *hydC017/hyd15* ovaries were completely rudimentary (without GCs) or contained stand-alone germline cysts (Figures 2E–G). Mutant gonads formed normally in embryos and larvae, but their germline degraded at later stages; only some GCs gave rise to cysts that produced a normal ECh. Nevertheless, the formed EChs also degraded during mid-oogenesis: only 1% of them developed into eggs. Thus, the *hyd* mutation affected GC viability at different stages of GC development, thereby causing the death of both early GCs (including stem cells) in larval ovaries and germarium and differentiated GCs in mid-oogenesis.

Besides reduced EChs, a small number of *hydC017/hyd15* ovaries also contained multi-nuclear EChs (Figure 2D and Supplementary Table 1). To determine whether such EChs were a consequence of excessive cystoblast mitosis, we performed immunostaining with an antibody to phosphorylated histone H3. The serine phosphorylation of histone H3 marks the onset of mitosis; therefore, any cyst in normal germaria contains no more than eight metaphase groups (Kiger et al., 2000). The phospho-H3 histone staining revealed that the mutant germline cysts contained significantly larger than normal of mitotic chromosomes. In particular, the number of metaphase groups inside one cyst reached 16 and 32, and the number of anaphases reached 32 and 64 (Figures 2H,I). As a result, both germaria and EChs looked abnormally enlarged. This phenotype was a consequence of additional mitoses in germline cysts and indicated abnormal cell cycle control when *hyd* was mutated.

### Germline-Specific Inhibition of *hyd* Expression Causes Massive Cell Death

The association of cell death with a decrease in the *hyd* activity was confirmed by our data from *hyd-*RNAi. For this experiment, we employed the *UAS/GAL4* system. To ectopically suppress the *hyd* gene expression in different ovary cells, we used the following tissue-specific drivers: *nanos*-GAL4 and *oskar*-GAL4 for GCs and *7023-GAL4*, *7024-GAL4* or *36287-GAL4* for somatic (follicular) cells. *UAS hyd-*RNAi strains and Oregon R were used as control.

Implementation of *hyd*-RNAi in follicular cells did not lead to any phenotypic changes in the ovaries, oogenesis abnormalities, or a decrease in fertility. Only a combination of *UAS-hyd*-RNAi with a germline-specific driver, *nanos-GAL4* or *oskar-GAL4*, resulted in massive cell death and ovarian atrophy, which were similar to the phenotypic manifestation of the *hyd* mutations under study.

#### Early GC Death in Nanos-GAL4/UAS-*hyd*-RNAi Ovaries

**FIGURE 3:**
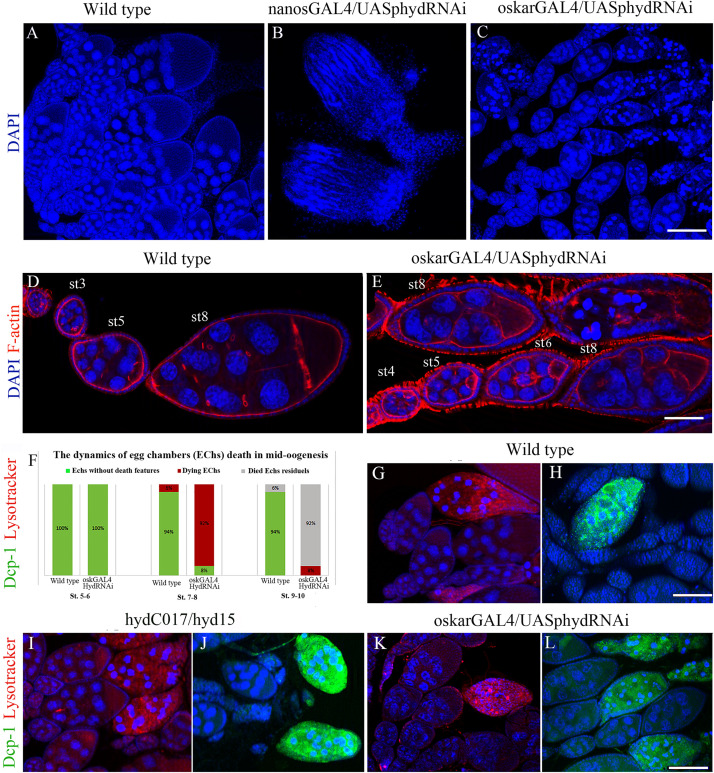
Effects of the *hyd* RNAi knockdown on ovarian morphology and oogenesis. **(A)** Wild-type ovaries with normal ovarioles and EChs. **(B)** Reduced (somatic) ovaries of *nanos-GAL4/UAS-hyd-*RNAi females, lacking germ cells. **(C)** Mid-stage oogenesis arrest and massive ECh degradation in an *oskar-GAL4/UAS-hyd*-RNAi ovary. **(D,E)** Comparison of ovariole and ECh morphology at early oogenesis stages between a wild-type **(D)** and *oskar-GAL4/UAS-hyd*-RNAi ovary **(E)**. **(F)** Diagram representing a comparison of the number of dying egg chambers between the wild-type and *oskar-GAL4/UAS-*hyd-RNAi ovaries during the middle oogenesis. No. of examined ovaries – 50, for each type. **(G–H)** Cytological detection of lysosomal activity and effector caspase Dcp-1 in wild type, *hydC015/hyd15* and *oskar-GAL4/UAS-hyd*-RNAi degrading EChs. Dying egg chambers of all types of ovaries demonstrate similar patterns of LysoTracker and anti-Dcp-1 staining: wild-type (**G,H**), *hydC017/hyd15* (**I,J**), *oskar-GAL4/UAS-*hyd-RNAi (**K,L**). DNA: DAPI (blue), lysosomes: LysoTracker (red), Dcp-1 staining: Alexa 488 (green), F-actin staining: phalloidin (red). Scale bars: 15 μm **(A–C)**, 3 μm **(D,E)**, 12 μm **(G–L)**.

*Nanos-GAL4* is expressed in primordial GCs starting from embryogenesis. Accordingly, in *nanos-GAL4/UAS-hyd*-RNAi females, GC death occurred before the adult stage. In adult females, almost all the germline turned out to be atrophied, and the formed ovaries consisted only of somatic cells (Figures 3A,B).

**FIGURE 4:**
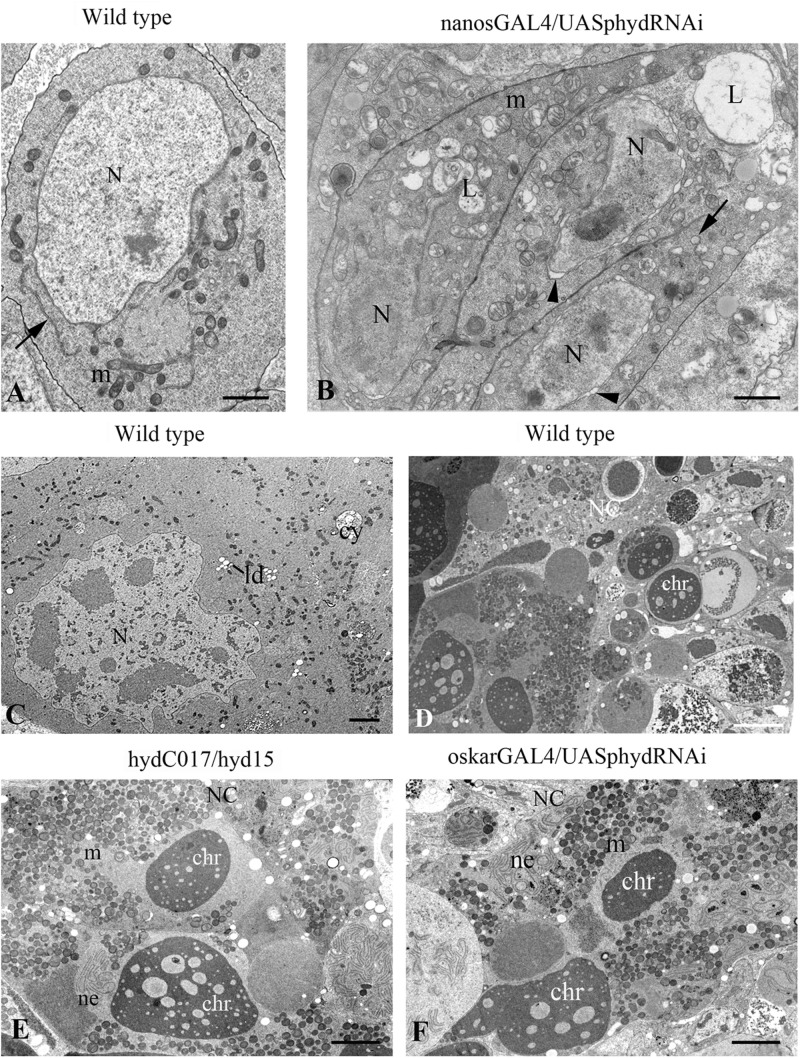
Ultrastructural features of germ cell death in *hyd*-RNAi ovaries. **(A–B)** Electron micrographs of primordial GCs in wild-type and *nanos-GAL4/UAS-hyd*-RNAi ovaries at the third instar larva stage. **(A)** A wild-type GC with morphology typical of this stage: nuclei with smooth contours, evenly dispersed chromatin, and distinct nucleoli; the cytoplasm contains round or oval mitochondria, endoplasmic-reticulum cisterns (ER cisterns), Golgi complexes, and abundant ribosomes. **(B)** GCs with signs of intracellular degradation: condensation of the nuclear matrix, curvature of the nuclear outline, nuclear envelope dilation (arrowheads), mitochondrial swelling, ER vacuolization (arrows) and numerous lysosomes. N, nucleus; m, mitochondrion; L, lysosome. Scale bar: 1 μm. **(C–F)** Signs of mid-oogenesis cell death in a wild-type, *hydC015/hyd15* and *oskar-GAL4/UAS-hyd*-RNAi ECh. **(C)** A segment of a mid-oogenesis ECh before cell death. Nurse cell ultrastructure without structural anomalies or signs of degradation. **(D–F)** Morphology of a dying ECh in a wild-type **(D)**, *hydC015/hyd15* **(E)**, and *oskar-GAL4/UAS-hyd*-RNAi **(F)**. Nurse cell cytoplasm contains condensed chromatin material, aggregates of nuclear-envelope fragments and clusters of mitochondria, numerous lysosomes. In the nurse cell area, there are of varying content present in this layer. NC, nurse cell; N, nucleus; m, mitochondrion; chr, chromatin; ne, nuclear envelope. Scale bar: 5 μm.

The *nanos-GAL4/UAS-hyd*-RNAi ovaries formed normally until third instar larva and at this stage were indistinguishable from the control under a light microscope. In contrast, electron microscopy uncovered multiple signs of intracellular degradation. They included condensation of the nuclear matrix, nuclear-envelope invaginations, dilation of organelles and an increased number of various cytoplasm vesicles, including multi-vesicular and myelin bodies, lysosomes and stand-alone autophagosomes (Figures 4A,B). Such structural changes are suggestive of decreased functional activity of the cells. These processes extended to the entire germline of *nanos-GAL4/UAS-hyd*-RNAi ovaries, inducing their atrophy and somatic gonad formation.

#### Mid-Oogenesis GC Death in *oskar-GAL4*/*UAS*-*hyd*-RNAi Females

The *oskar-GAL4* driver is activated at stages 3–4 of oogenesis; accordingly, in *oskar-GAL4/UAS-hyd*-RNAi females, GC death occurred in mid-oogenesis (Figure 3C). Before dying, after stage 4 or 5, EChs failed to grow normally. Even though the *oskar-GAL4/UAS-hyd*-RNAi EChs continued developing, their size was much smaller than that in normal chambers at the same stage (Figures 3D,E and Supplementary Figure 1). EChs, growing slowly, accumulated in ovarioles, which consequently became abnormally long. In normal ovarioles, only 4–6 EChs of different developmental stages, including late oogenesis, are usually observed. In *oskar-GAL4/UAS-hyd*-RNAi females, ovaries consisted of 6–9 EChs that slowly developed mainly up to the eighth stage. The delay in development ended with mid-oogenesis arrest and massive death of GCs. As a result, *oskar-GAL4/UAS-hyd*-RNAi ovaries contained, instead of vitellogenic EChs, a mass of degrading material. Only a small proportion, ∼8% of EChs, progressed to stages 9–10 but then died at the beginning of late oogenesis. Therefore, the *hyd*-RNAi induced by the *oskar-GAL4* driver caused ECh growth retardation, which led to their complete degradation (100%).

Mid-stage death is a characteristic feature of *Drosophila* oogenesis and allows to save energy and resources eliminating defective or unnecessary EChs. Dying EChs are easily identified by abnormal condensation and subsequent fragmentation of chromatin, which are features of apoptotic cell death (Greenwood and Gautier, 2005; Sarkissian et al., 2014). In addition to apoptosis, autophagy is also involved in mid-oogenesis cell death (Bolobolova et al., 2020). Recent studies have convincingly shown that ubiquitin proteasomal machinery is important for the regulation of cell death pathways and that many E3 ubiquitin ligases take part in this process. To determine how E3 ligase Hyd can be associated with mid-oogenesis death, we investigated this process in more detail by means of the main cytological markers of apoptosis and autophagy together with electron microscopy.

To detect autophagy, we utilized LysoTracker, an acidophilic dye that marks lysosomes and autophagosomes. A LysoTracker signal was detected in all *oskar-GAL4/UAS-hyd*-RNAi ovarioles, but its appearance coincided with the beginning of nuclear condensation, same as in the controls (Figures 3F,G). To verify the apoptotic events, we used an antibody recognizing effector caspase Dcp-1: a general apoptosis marker in *Drosophila* (Sarkissian et al., 2014). Immuno-fluorescence staining suggested that anti-Dcp-1 antibodies were incorporated into degenerating EChs of *hyd*-RNAi ovaries, and their pattern was indistinguishable from that in the control (Figures 3H,I).

We conducted an electron microscopic analysis to clarify the features of the cell death and to identify a possible pathological change in the germline before the death. Ultrastructural analysis of *oskar-GAL4/UAS-hyd*-RNAi imago ovaries showed that EChs underwent cell death at stages 7–8/9, as in the wild type. Moreover, some oocytes in the dying chambers contained stand-alone vitelline granules, which mean the onset of vitellogenesis. Before these stages, substantial abnormalities in GC ultrastructure were not detectable. The ECh degeneration was accompanied by specific structural transformations similar to those in the wild type (Giorgi and Deri, 1976). The nurse cell cytoplasm and nucleus featured increased electron density, and nuclear chromatin formed condensed masses. With further degeneration, the nuclear envelope in the nurse cells broke down, and nuclear material was released into the cytoplasm. Numerous fragments of nuclear membranes formed aggregates in the nurse cell cytoplasm with a specific parallel orientation in tracts. Most mitochondria combined into clusters. The cytoplasm contained large vacuoles of varying content and electron density (Figures 4C,D,F). The same cell death manifestations were observed in the *hydC017/hyd15* EChs (Figure 4E). All these events are characteristic signs of mid-oogenesis cell death, as described elsewhere (Giorgi and Deri, 1976).

Thus, the RNAi knockdown of the *hyd* gene did not cause anomalies in EChs’ ultrastructure before their death. The ECh degradation occurred at the same stage of oogenesis as in the wild type, did not differ morphologically and had signs of both apoptosis and autophagy. This means that the GC death in *oskar-GAL4/UAS-hyd*-RNAi ovaries proceeds via the canonical *Drosophila* mid-oogenesis pathway.

## Discussion

This study showed that Hyd is critical for GCs’ development and egg formation in *Drosophila*. The lack of this protein led to massive cell death and ECh degradation and, as a minor manifestation, to GC overproliferation as a consequence of additional mitoses. The observed effect was germline specific and independent of a somatic environment.

As an E3 ubiquitin ligase, Hyd is one of the key components of the ubiquitination system, which is important for the regulation of activities of proteins, their cellular functions and proteasomal degradation (Bhoj and Chen, 2009; Shearer et al., 2015) Analysis of protein–protein interactions by a yeast two-hybrid assay has allowed to identify more than 50 proteins that can potentially interact with Hyd in ovaries^1^ (Supplementary Table 1). Hyd interactors can be both substrates for ubiquitination and proteins that cooperate with Hyd to form functional complexes. The variety of these proteins determines the multiple functions of Hyd in *Drosophila* oogenesis; therefore, its dysfunction or loss has dramatic downstream consequences.

### Hyd Is Essential for the Maintenance of Germline Viability

Our findings indicate that Hyd is especially important for GC survival. Its lack induced the death of both early GCs in the larval ovaries and differentiated ones in mid-oogenesis.

According to our results, the death of Hyd-deficient GCs in *nanos-GAL4/UAS-hyd*-RNAi ovaries begins in late third instar larvae. The ovaries at this stage contain primordial germ cells (PGCs) that terminate to divide and specialize in two directions: several cells interact with a newly formed niche and become germline stem cells, whereas the remaining cells begin to differentiate and become cystoblasts (Gancz et al., 2011; Kahney et al., 2019; Hinnant et al., 2020). Germline differentiation factor Bam governs both stem cell maintenance and the differentiation of their progeny: Bam expression is inhibited in stem cells (where differentiation is prohibited) and activated in cystoblasts (where differentiation is promoted) (Ji et al., 2017; Mathieu and Huynh, 2017; Kahney et al., 2019). Bam over-expression in late third instar larva ovaries causes massive GC death and the formation of completely somatic gonads (Ohlstein and McKearin, 1997; Chen and McKearin, 2003). We noticed a similar phenotype when Hyd was either mutated or knocked down.

GC death at this stage of development is also associated with several intrinsic factors. It has been documented that such an effect is exerted by a strong mutation or knockout of any of the following genes: *ote* (coding for a nuclear lamina protein) (Barton et al., 2013), *mcm10* (its product acts in DNA replication) (Reubens et al., 2015), γ*-tubulin* (encodes the main component of the peri-centriolar material) (Tavosanis and Gonzalez, 2003) and *vasa* (its product has a multi-functional role in the germline) (Styhler et al., 1998). The same is true for over-expression of Dmp53, a homolog of mammalian p53 (Bakhrat et al., 2010). Nonetheless, almost all proteins encoded by these genes are important even before the late third instar stage. Only Ote has been shown to start functioning at this stage, and Ote is necessary for the progression of differentiation. In *ote* mutants, GCs are not able to differentiate and to die (Barton et al., 2013). On the other hand, stem cells are not susceptible to Ote loss and persist in adult ovaries (Barton et al., 2013), in contrast to the Hyd deficiency phenotype. Accordingly, we assume that Bam or components of Bam signaling are the most likely partners of Hyd at the very beginning of oogenesis, when the fate of PGCs is determined: to be stem cells or differentiate.

Massive death of germ cells also occurred in *oskar-GAL4/UAS-hyd*-RNAi ovaries through middle oogenesis. EChs containing Hyd-deficient GCs did not progress through the oogenesis mid-stage checkpoint and degraded completely in early vitellogenesis. It is known that this checkpoint is activated in response to adverse stimuli or physiological and developmental disorders (Pritchett et al., 2009; Jenkins et al., 2013; Peterson et al., 2015). GC death at this stage is tightly regulated both positively and negatively with the help of a genetic network that includes, in particular, genes responsible for the ubiquitination mechanism. Dysregulation or loss of function of these genes induces disturbances in cell death manifestations (Hou et al., 2008; Bergmann, 2010; Peterson et al., 2015). One of these abnormal manifestations is associated with cell death enhancement in mid-oogenesis. In particular, such a phenotype is observed when the genes encoding inhibitors of apoptosis (members of the IAP protein family) Bruce and Diap are suppressed (Rodriguez et al., 2002; Hay and Guo, 2006; Hou et al., 2008). However, the typical feature of the Hyd-deficient-ovary phenotype is not only mid-oogenesis arrest and increased cell death but also the preceding slowdown in ECh development and growth. Moreover, degrading EChs do not morphologically differ from the wild type and have signs of autophagy and apoptosis (these are normal).

These phenotypic features emerge in response to nutrient starvation or a reduction in insulin/TOR signaling (Drummond-Barbosa and Spradling, 2001; Barth et al., 2011; Pritchett and McCall, 2012). The insulin/TOR signaling network is a highly conserved mechanism responsible for cell and tissue growth. It acts as a sensor of nutrient availability to promote cell metabolism, growth and proliferation. In *Drosophila* oogenesis, the insulin/TOR axis is critical for GC development and oocyte maturation. In the presence of some disorders of this mechanism, EChs’ growth is delayed, and they cannot enter energy-intensive vitellogenesis and hence degenerate (LaFever et al., 2010; Laws and Drummond-Barbosa, 2017; Jeong et al., 2019). We believe that Hyd either interacts with one of insulin/TOR targets or acts upstream by regulating the factors responsible for the biosynthesis in growing EChs.

Currently, there are no experimental data supporting the interaction of *Drosophila* Hyd with components of the insulin/TOR signaling network. Nonetheless, lately, it was reported that Hyd’s human homolog, EDD, is involved in the regulation of mTOR (TORC1) via ubiquitination for proteasomal degradation of its target PP2Ac (TORC1-associated ɑ4 phosphoprotein). Increased EDD expression in human breast cancer cells *in vitro* promotes TORC1 signaling, which activates the synthesis of an anti-apoptotic protein promoting drug resistance. On the contrary, a knockdown of EDD arrests cancer cell proliferation decreases their viability and increases apoptosis (MacDonald et al., 2019).

According to Flybase and DroID, the Hyd protein interacts with TOR signaling pathway proteins (Pdk1 and RagC) and with many factors necessary for the synthesis and modification of proteins and RNA and for Golgi and endoplasmic-reticulum functioning (Supplementary Table 2). Nonetheless, several studies should be conducted to determine which potential substrate interacts with E3 ubiquitin kinase Hyd *in vivo* to ensure germ cell survival.

### One of Hyd Functions in Oogenesis Is Associated With the Control of Germ Cell Divisions

Some studies on the somatic tissue in imaginal discs have shown that Hyd is a negative regulator of Hh signaling. Hyd-deficient flies display eye disc overgrowth due to increased Hh activity (Mansfield et al., 1994; Lee et al., 2002; Hariharan and Bilder, 2006). In oogenesis, Hh participates in the control of germ cell proliferation through a somatic microenvironment: this protein is produced by a niche of stem germ cells to balance stem cell renewal and differentiation (Lu et al., 2015; Huang et al., 2017; Lai et al., 2017). One would expect that Hyd and Hh also interact in this context. According to our data, however, Hyd is necessary to control the number of cystoblast divisions, not stem cells, and unlike Hh, the Hyd protein can act autonomously in the germline. Therefore, we believe that the role of Hyd in female GC divisions is not mediated by Hh signaling either.

An extra round of cystoblasts’ mitosis, as in *hyd* mutants, is also observed during dysregulation of the timely degradation of mitotic cyclins: cyclins A, B and E (Lilly et al., 2000; Wang and Lin, 2005; Chen et al., 2009). For instance, overexpression of genes encoding these proteins or a deletion of the CycA or CycB destruction box gives rise to one, less often two, extra rounds of mitotic division, resulting in 32- and 64-cell cysts (Jacobs et al., 1998; Doronkin et al., 2003). The degradation of cyclins is implemented mainly through ubiquitin-proteasome-dependent proteolysis; hence, its failures also prevent timely termination of mitosis (Ji et al., 2017; Chen et al., 2018; Hinnant et al., 2020). In *Drosophila* oogenesis, mutations of genes playing a part in the ubiquitination system induce an ‘extra round of mitosis’ phenotype, as in a *hyd*-deficient background. In particular, such a phenotype is observed after mutations of genes encoding subunits of the SCF complex (Skp1, Cullins, and F-box proteins), which is an E3 ubiquitin ligase. In these cases, cyclin E accumulation, entry into the S phase and doubling of the cell number are observed (Ohlmeyer and Schüpbach, 2003). The same phenotype is generated by mutations in the *effete* gene (encoding E2 ubiquitin-conjugating enzyme UbcD1) (Lilly et al., 2000), *encore* (coding for a protein interacting with the proteasome and Cyclin E) (Hawkins et al., 1996; Ohlmeyer and Schüpbach, 2003) or *slmb* (coding for an E3 ubiquitin ligase) (Muzzopappa and Wappner, 2005).

Recent research has shown that the stabilization of CycA expression during mitotic cystoblast divisions is controlled by Bam, which acts together with deubiquitinase Otu (Ji et al., 2017). Earlier in this article, we suggested that Hyd may be an antagonist of Bam signaling in early oogenesis, at the beginning of PGC differentiation. Perhaps their interplay continues during germline cyst divisions in the germarium, where Hyd, in contrast of Bam, is required for downregulation of cyclins and for timely ending of germ cell mitosis.

It is currently difficult to determine what other factors may be associated with Hyd in this context. The list of potential interactors contains several proteins responsible for cell cycle control (Supplementary Table 2). On the other hand, their participation in proliferation has not been demonstrated experimentally. Therefore, additional original data are needed to resolve this issue.

In this article, we describe a previously unknown function of *Drosophila* Hyd in oogenesis and germ cell development. We demonstrated that Hyd is essential for timely regulation of germline cyst mitosis and for the maintenance of germ cell viability. Because Hyd is an E3 ubiquitin ligase, it contributes to cellular processes through ubiquitination of its substrate proteins. In the future, it is necessary to identify these proteins and determine their germline-specific functions in order to understand which signaling pathways and mechanisms mediate Hyd’s involvement in oogenesis control.

## Data Availability Statement

The original contributions presented in the study are included in the article/Supplementary Material, further inquiries can be directed to the corresponding author.

## Author Contributions

ND, YG, and SF designed and conducted the experiments, analyzed the data, wrote and prepared the manuscript. EUB provided obtaining and analysis of electron microscopic data. EMB analyzed the data. All authors contributed to the article and approved the submitted version.

## Conflict of Interest

The authors declare that the research was conducted in the absence of any commercial or financial relationships that could be construed as a potential conflict of interest.

## Footnotes

1 http://www.flybase.org

## Supplementary Material

Supplementary Table 1 ‘Extra round of mitosis’ phenotype manifestations.

Supplementary Table 2 Hyd interacting proteins.

Supplementary Figure 1 Egg chamber sizes reduction in *oskar-GAL4/UAS-hyd*-RNAi females. The measurement of the length and width of the egg chambers was carried out using LSM5 Image Browser (Zeiss). The length and width of the egg chambers in *oskar-GAL4/UAS-hyd*-RNAi ovaries are statistically valid less than in the wild type (Oregon). Data represent mean ± s.d., Student’s *t*-test; ****P* < 0.001; *n* = 10 egg chambers per condition. Diagrams were generated and statistical analyses performed in Prism (GraphPad).
