# Supplementary material for: Loss of Drosophila E3 Ubiquitin Ligase Hyd Promotes Extra Mitosis in Germline Cysts and Massive Cell Death During Oogenesis
Source: Front Cell Dev Biol. 2020 Nov 9;8:600868. doi: 10.3389/fcell.2020.600868 (PMC7680892; doi:10.3389/fcell.2020.600868)
Supplement: Supplementary Table 1 — ‘Extra round of mitosis’ phenotype manifestations. [file Data_Sheet_1.PDF]

Table S1. 'Extra round of mitosis' phenotype manifestations (partially reduced ovaries)

Genotype	% of ovaries with extra-mitosis in germarium	average of germaria with extra-mitosis per ovary	% of ovaries with multinuclear egg chambers	average of multinuclear egg chambers per ovary	total number of ovaries examined
hyd$^+$/*hyd*$^+$ (Oregon)	0	0	0	0	80
hydC017/hyd15	32%	2, 6±1,3	41%	3,2±1,7	72
