# Supplementary material for: Loss of Drosophila E3 Ubiquitin Ligase Hyd Promotes Extra Mitosis in Germline Cysts and Massive Cell Death During Oogenesis
Source: Front Cell Dev Biol. 2020 Nov 9;8:600868. doi: 10.3389/fcell.2020.600868 (PMC7680892; doi:10.3389/fcell.2020.600868)
Supplement: Supplementary Table 2 — Hyd interacting proteins. [file Data_Sheet_2.PDF]

Table S2. Hyd interacting proteins

Symbol	Fly gene (Flybase ID)	GO Biological process
**Cell cycle**		
Pdk1	FBgn0020386	regulation of cell growth
Sgg	FBgn0003371	positive regulation of mitotic metaphase/anaphase transition,
Mts	FBgn0004177	mitotic cell cycle
Myc	FBgn0262656	cell proliferation, regulation of cell cycle
CG14262	FBgn0039503	SCF ubiquitin ligase complex
Wee1	FBgn0011737	mitotic cell cycle checkpoint
Rad9	FBgn0025807	DNA replication checkpoint, DNA damage checkpoint
Mkp3	FBgn0036844	regulation of cell proliferation, negative regulation of MAPK cascade
Ci	FBgn0004859	Transcription / Cell division and chromosome partitioning, positive regulation of G1/S transition of mitotic cell cycle
Gmd	FBgn0031661	Notch signaling pathway, regulation of stem cell division
Fzr2	FBgn0034937	exit from mitosis, anaphase-promoting complex-dependent catabolic process, positive regulation of ubiquitin protein ligase activity
**Apoptosis**		
Pdk1	FBgn0020386	negative regulation of apoptotic process
Myc	FBgn0262656	regulation of apoptotic process
CG5059	FBgn0037007	positive regulation of apoptotic process, regulation of programmed cell death, mitochondrial outer membrane permeabilization
Corp	FBgn0030028	p53 binding, negative regulation of intrinsic apoptotic signaling pathway in response to DNA damage
Diap1	FBgn0260635	negative regulator of apoptosis
**Autophagy**		
Pdk1	FBgn0020386	mTOR signaling pathway
Mts	FBgn0004177	autophagy
Myc	FBgn0262656	regulation of autophagy
Wdr24	FBgn0027518	positive regulation of macroautophagy
RagC-D	FBgn0033272	positive regulation of TOR signaling, cellular response to starvation, regulation of autophagy
Tsp	FBgn0031850	cell adhesion mediated by integrin, Phagosome
Atg101	FBgn0030960	autophagosome assembly, autophagy, positive regulation of macroautophagy, larval midgut cell programmed cell death
**UPS (ubiquitin proteolysis system)**		
Sgg	FBgn0003371	protein phosphorylation, regulation of proteolysis
CG14262	FBgn0039503	SCF ubiquitin ligase complex,
Vhl	FBgn0041174	ubiquitin-protein transferase activity ;ubiquitin protein ligase activity
Rpt6	FBgn0020369	proteasome regulatory particle, Posttranslational modification, protein turnover, chaperones
Rpt1	FBgn0028687	proteasome regulatory particle, Posttranslational

		modification, protein turnover, chaperones
Prosbeta2R2	FBgn0037296	proteasome core complex
Mib2	FBgn0086442	ubiquitin-protein transferase activity
Jon44E	FBgn0001285	proteolysis
Usp10	FBgn0052479	protein deubiquitination
CG30496	FBgn0050496	negative regulation of protein ubiquitination
CG15800	FBgn0034904	ubiquitin-dependent protein catabolic process,SKP1 component
**Others**		
Vha36-1	FBgn0022097	proton transport, Phagosome
Trf	FBgn0010287	positive regulation of transcription from RNA polymerase II promoter
Thoc7	FBgn0035110	mRNA processing
Fd59A	FBgn0004896	transcription factor activity
Rexo5	FBgn0286051	RNase T/DNA polymerase III
PIG-T	FBgn0030035	endoplasmic reticulum
O-fut2	FBgn0027791	endoplasmic reticulum, Golgi apparatus, regulation of transcription
Lectin-46Ca	FBgn0040093	protein localization, extracellular space
Hsp70Aa	FBgn0013275	response to hypoxia, response to unfolded protein
Hsp22	FBgn0001223	Posttranslational modification, protein turnover, chaperones, oxidative stress response, mitochondrion
Sema1a	FBgn0011259	positive regulation of cell migration
Sals	FBgn0051374	actin filament binding
Osk	FBgn0003015	regulation of mRNA stability, positive regulation of translation
Gs1	FBgn0001142	phagocytosis, mitochondrion organization
Dph1	FBgn0036194	phagocytosis
CG9596	FBgn0031832	tRNA methylation, lateral inhibition
CG5541	FBgn0030603	integral component of membrane,neuromuscular synaptic transmission, long-term memory
CG30350	FBgn0050350	protein peptidyl-prolyl isomerization, protein folding
CG33523	FBgn0053523	neurogenesis, ER membrane
CG3358	FBgn0033117	Mg-dependent DNase
Brf	FBgn0038499	regulation of transcription from RNA polymerase III promoter
Atms	FBgn0010750	transcription elongation from RNA polymerase II promoter
Arp1	FBgn0011745	cytoskeleton organization, microtubule-based movement
Akirin	FBgn0082598	positive regulator of the immune deficiency (Imd) pathway
CG4004	FBgn0030418	MADF domain
CG3526	FBgn0040355	zinc ion binding
CG31835	FBgn0051835	zinc ion binding
CG32295	FBgn0260480	unknown
CG30412	FBgn0050412	unknown
CG1561	FBgn0030317	unknown
CG12123	FBgn0030039	unknown
