# Supplementary material for: Loss of Drosophila E3 Ubiquitin Ligase Hyd Promotes Extra Mitosis in Germline Cysts and Massive Cell Death During Oogenesis
Source: Front Cell Dev Biol. 2020 Nov 9;8:600868. doi: 10.3389/fcell.2020.600868 (PMC7680892; doi:10.3389/fcell.2020.600868)
Supplement: Supplementary Figure 1 — Egg chamber sizes reduction in oskar-GAL4/UAS-hyd-RNAi females. The measurement of the length and width of the egg chambers was carried out using LSM5 Image Browser (Zeiss). The length and width of the egg chambers in oskar-GAL4/UAS-hyd-RNAi ovaries are statistically valid less than in the wild type (Oregon). Data represent mean ± s.d., Student’s t-test; ***P < 0.001; n = 10 egg chambers per condition. Diagrams were generated and statistical analyses performed in Prism (GraphPad). [file Data_Sheet_3.PDF]

Egg chamber sizes reduction in *oskar-GAL4/UAS-hyd*-RNAi females

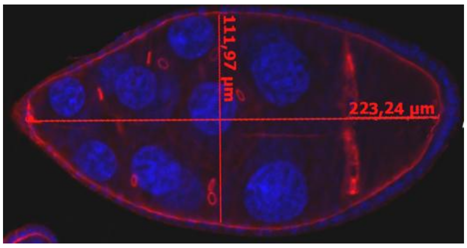

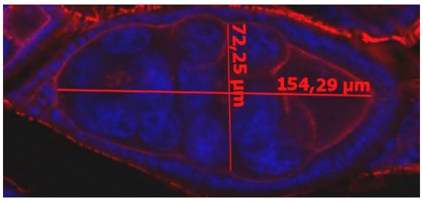

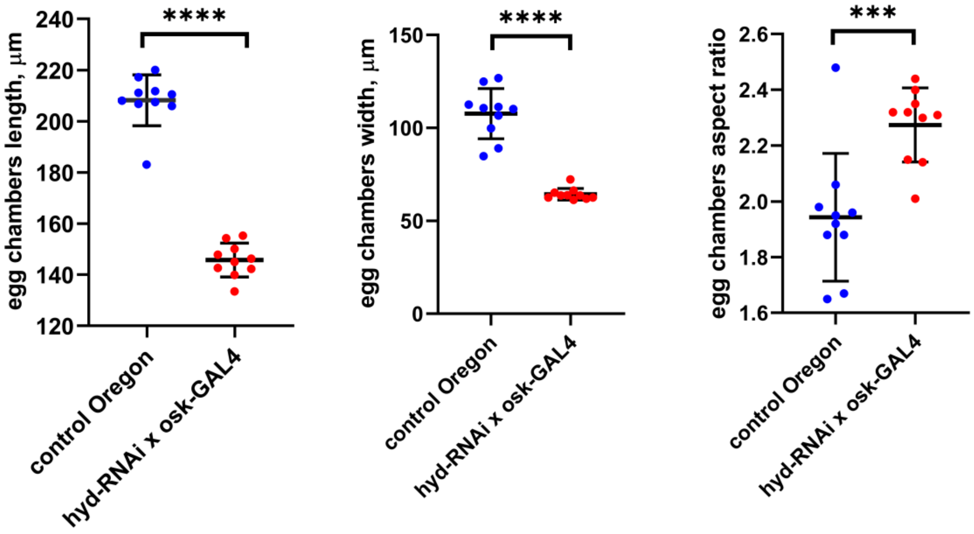

The measurement of the length and width of the egg chambers was carried out using LSM5 Image Browser (Zeiss).

The length and width of the egg chambers in *oskar-GAL4/UAS-hyd*-RNAi ovaries are statistically valid less than in the wild type (Oregon). Data represent mean±s.d., Student's t-test; ***P<0.001; n=10 egg chambers per condition. Diagrams were generated and statistical analyses performed in Prism (GraphPad).
